# Assessment of drug levels as a biomarker in rheumatoid arthritis

**DOI:** 10.1016/j.ero.2025.11.026

**Published:** 2025-12-26

**Authors:** Tsutomu Takeuchi, Hiroya Tamai, Yuko Kaneko

**Affiliations:** 1 Depertment of Rheumatology and Applied Immunology/Rheumatology and Clinical Immunology, Saitama Medical University, Moroyama, Iruma, Saitama, Japan; 2 Division of Rheumatology, Department of Internal Medicine, Keio University, Tokyo, Japan

## Abstract

In this viewpoint, we focused on 2 examples of the assessments of drug levels as a biomarker in rheumatoid arthritis (RA), based on our experiences in longitudinal studies and clinical trials. Among a long list of potential biomarkers in rheumatology, the measurements of drug levels for the efficacy and toxicity of the drugs have been extensively investigated. One example is the levels of methotrexate (MTX)-polyglutamates (PGs), and the second one is that of biological disease-modifying antirheumatic drugs (DMARDs) in patients with RA. Recently, meta-analyses and population pharmacokinetics-pharmacodynamics studies have been published, confirming the previous reports. After these publications, we experienced the new information about the measurements of MTX-PGs in a prospective multicenter clinical trial. In addition, we also experienced the measurements of drug levels of a unique bDMARD, ozoralizumab, a bispecific Variable domain of Heavy chain of Heavy chain antibody (VHH) molecule, which is directed against TNF*α* and albumin in clinical trials. We introduced our experiences and explored the viewpoint of the measurements of drug levels as a biomarker in RA.

## VIEWPOINT

Medical treatment is a key part of the strategy for treating rheumatic and musculoskeletal diseases. One of the best examples is disease-modifying antirheumatic drugs (DMARDs) in the treatment algorithm for rheumatoid arthritis (RA) [1,2]. During the early development of DMARDs, it was widely recognized that the therapeutic effect of most drugs might be slow to manifest and that monitoring drug levels could allow adjustments to be made to the dose or intervals to achieve better and faster outcomes [3]. In addition, it can provide information about the safety of the drugs. Monitoring of drug levels can improve our understanding of the mechanism of action of drugs and is expected to provide a rationale in guiding treatment [4]. However, stringent criteria must be met for them to be used as biomarkers or laboratory markers in clinical practice [5]. When we consider the lists of biomarkers for diagnosis, staging, prognosis, and predicting and monitoring clinical response (Table 1), several potential biomarkers for therapeutic drug monitoring (TDM) emerge. Although these may support our understanding and interpretation of results, one might argue that they still lack generalizability, reliability, reproducibility, and cost-effectiveness [5]. For biopharmaceuticals, the EULAR concluded that proactive use of TDM is not recommended, but that reactive TDM could be considered in certain clinical situations [6,7].

**Table 1:** Potential blood biomarkers in rheumatology

1. Acute phase reactants: ESR, CRP, ferritin, amyloid A, procalcitonin, etc. 2. Antibody/autoantibody: IgG, IgG4, IgM, IgA, anti-dsDNA, anti-RNP/Sm, anti-phospholipid, anti-B2GP1, anti-neutrophil cytoplasmic (ANCA), anti-tRNA synthase (ARS), anti-TIF1γ, anti-MDA5, anti-topoisomerase 1, anti-centromere, anti-SSA/SSB, anti-CCP, RF, IgG-RF, etc. 3. Other immunology/inflammation: immune complexes, sIL2R, CH50, C3, C4, etc. 4. Gene/gene products: MEFV gene, NLRP3, NOD2, TNFSF1A, HLA-B27, HLA-DR-shared epitope, etc 5. Bone metabolism biomarkers: NTX, CTX, osteocalcin, BAP, etc. 6. Tissue/pathogenesis biomarker: amylase (salivary, pancreas), creatinine kinase (CK, CK-MM, CK-MB), cystatin C, MMP-3, KL-6, SP-D, troponin, ADAMTS13, etc. 7. Drug monitoring for csDMARDs: MTX-PGs, ciclosporin, tacrolimus, mycophenolate, etc 8. Drug monitoring for bDMARDs: - Target levels:IL-6, TNFα, sIL-6R, B cell, etc. - bDMARD levels:infliximab, etanercept, adalimumab, rituximab, tocilizumab, golimumab, certolizumab-pegol, sarilumab, ozoralizumab, etc. - Anti-drug antibody 9. Safety: β-d-glucan (pneumocystis jirovecii pneumonia), TPMT (azathioprine), NUDT15 (azathioprine), etc.

ADAMTS13, A disintegrin and metalloproteinase with a thrombospondin type 1 motif, member 13; ANCA, antineutrophil cytoplasmic antibody; ARS, anti-transfer RNA synthase; BAP, bone alkaline phosphatase; bDMARD, biologic disease-modifying antirheumatic drug; CCP, cyclic citrullinated peptide; CK, creatinine kinase; CK-MB, creatinine kinase-myocardial band; CK-MM, creatinine kinase-muscle type; CRP, C-reactive protein; csDMARD, conventional synthetic disease-modifying antirheumatic drug; CTX, C-terminal telopeptide; ESR, erythrocyte sedimentation rate; HLA-B27, human leukocyte antigen B27; HLA-DR, human leukocyte antigen DR; IgA, immunoglobulin A; IgG, immunoglobulin G; IgG4, immunoglobulin G4; IgG-RF, immunoglobulin G rheumatoid factor; IgM, immunoglobulin M; IL-6, interleukin-6; KL-6, Krebs von den Lungen-6; MDA5, melanoma differentiation-associated gene 5; MEFV, Mediterranean fever; MMP-3, matrix metalloproteinase-3; MTX-PG, methotrexate polyglutamate; NLRP3, NLR family pyrin domain-containing 3; NOD2, nucleotide-binding oligomerization domain-containing protein 2; NTX, N-terminal telopeptide; NUDT15, Nudix hydrolase 15; RF, rheumatoid factor; sIL-6R, soluble interleukin-6 receptor; SP-D, surfactant protein D; SSA, Sjörgren’s syndrome-related antigen-A; SSB, Sjörgren’s syndrome-related antigen-B; TIF1γ, transcriptional intermediary factor 1-gamma; TNFSF1A, tumor necrosis factor superfamily member 1A; TNFα, tumor necrosis factor alpha; TPMT, thiopurine methyltransferase.

In this article, we picked up 2 examples for considering the monitoring of drug levels as a biomarker in patients with RA, primarily based on our experiences. The first example is MTX-polyglutamate (MTX-PG) concentrations in red blood cells (RBCs) in RA. Recently, the meta-analysis and the population pharmacokinetics (PKs)-pharmacodynamics (PDs) of measuring MTX-PG concentrations in RBCs confirmed that the higher MTX-PG concentrations correlate with the lower disease activity in RA, juvenile idiopathic arthritis [8], and psoriasis [8,9]. After this meta-analysis and population PK-PD had been published, we added new information about the erythrocyte MTX-PG concentrations in a prospective interventional clinical trial for MTX-naïve RA patients, who initiated MTX [10,11]. In these experiences and other personal communications for measuring erythrocyte MTX-PG concentrations, we explored the viewpoint for the assessment of erythrocyte MTX-PG concentrations for considering the efficacy and toxicities of oral MTX treatment in RA. The second example is the case of new anti-TNF biologics, ozoralizumab, in RA. The structure of this molecule is unique, so that the demonstration of the drug concentrations and population PKs may impart a new perspective to our understanding of the rapid and sustained control of the disease activity by this molecule [12].

## MTX-PG

Systematic review of drug monitoring of MTX as well as population PKs of MTX drug levels has been reported in high-dose MTX therapy in oncology [13,14]. Although MTX itself is rapidly metabolized from the circulation, MTX-PGs may be a stable biomarker even in the oncology with chronic conditions such as maintenance therapy in patients with childhood acute lymphoblastic leukemia [15].

Lower dose pulse treatment of methotrexate (MTX) is the anchor strategy for RA management [1,2]. Rapid escalation and higher-dose regimens are recommended to achieve the treatment target within week 24 after initiating MTX in the management of patients with RA [[16], [17], [18], [19]]. Maximum tolerable doses may differ among individuals, regions, and countries. Measuring the blood concentrations of MTX and its metabolites, particularly MTX-PGs within RBCs, may provide insight into the efficacy and toxicities of MTX in the individual patient or a group of patients. Two clinical studies of MTX-naïve early patients with RA who started MTX reported their MTX-PG concentrations in prospective longitudinal cohorts using the same LC techniques. The first study showed the MTX-PG concentrations in Albany, NY, between late 2002 and 2004 [20,21]. At this time, the MTX dose was lower than the current clinical practice, with the dose being increased cautiously until a therapeutic response was achieved [20]. It took 12 weeks or longer to achieve a concentration of 60 nmol/L, which may be an efficacious cutoff point (Albany study, *n* = 47) [21]. We experienced the measuring MTX-PG concentrations of Japanese patients with RA in a multi-center longitudinal cohort (MAGIK study, *n* = 79) [22], showing that MTX-PG concentrations in the MAGIK study were higher than those reported in Albany [20,21]. Although the MTX dose and escalation speed were comparable between the MAGIK study in Japan and the Albany study in the USA, the patient backgrounds were different in BMI (21.9 in the MAGIK study vs 28.6 in the Albany study), the folic acid supplementations, and so on. This raised a question whether body weight/BMI, folic acid supplementation, and other covariates at baseline may be the factors in explaining the difference in MTX-PGs.

Accumulated evidence now demonstrates that higher MTX-PG concentrations in RBCs are associated with lower disease activity in the longitudinal cohort at the group level [[21], [22], [23]]. Van de Rotte et al [23] showed in 2 longitudinal derivation and validation cohorts that an increase in MTX-PG concentrations in RBCs was associated with a decrease in DAS28 over 9 months after initiating MTX in patients with RA. Recently, meta-analysis and PK-PD modeling for the relationship between concentrations of MTX-PGs in RBCs and efficacy and toxicity in RA and other immune-mediated inflammatory diseases were published. In the meta-analysis, 25 clinical studies/trials, which satisfied the inclusion criteria, were analyzed [8]. They concluded that higher concentrations of erythrocyte MTX-PGs were associated with lower disease activity in RA, juvenile idiopathic arthritis, and psoriasis. Furthermore, PKs and PDs modeling of MTX-PGs in RBCs from 3 longitudinal cohorts, consisting of 395 patients and 3401 MTX-PG concentrations in a large cohort of patients with RA, elegantly demonstrated that MTX-PG3-5 levels in RBCs were associated with clinical response and that smoking, comedications, BMI, and disease activity at baseline were identified as the independent covariates with the response [9]. After this meta-analysis and population PK/PD modeling were published, we added new information on measuring erythrocyte MTX-PGs in a prospective clinical trial, which recruited 300 patients with RA, starting MTX [10,11]. In this clinical trial involving Japanese, Taiwanese and Korean MTX-naïve patients with RA who were scheduled to start taking MTX increased the dose to the maximum tolerable dose, aiming to achieve Simplified Disease Activity Index (SDAI) remission at weeks 24 as the treatment target (approved maximum dose: Japan 16 mg/wk, Taiwan and Korea 25 mg/wk, folic acid 10 mg/wk in Japan and Taiwan, and 1 mg/d in Korea) [10]. Patients who failed to achieve SDAI remission at 24 weeks after scheduled dose escalation were randomized into 2 groups: 1 continued with the same MTX dose, whereas the other had their MTX dose reduced and initiated adalimumab. Those who achieved SDAI remission continued with the same MTX dose [10]. In this trial, we planned to measure erythrocyte MTX-PG concentrations at weeks 0, 4, 8, 12, 24, 36, and 48, using a liquid chromatography-tandem mass spectrometry-based assay [11]. The mean concentrations of total MTX-PGs increased with an increasing dose of MTX and continued to elevate for another 12 weeks after the maximum tolerable dose was fixed. At week 24, total MTX-PG concentrations were 110.5 nmol/L with the dose of MTX 12.6 mg/wk (0.23 mg/kg/wk). This study confirmed that higher erythrocyte MTX-PG concentration is an independent factor for lower disease activity, supporting the meta-analysis and population PK-PD. In addition, the higher total MTX-PGs were related to the higher hepatotoxicity. Finally, MTX-PG concentration was significantly elevated by lower estimated glomerular filtrations, serum albumin, and body mass index [11]. In post hoc multivariable analysis, the factors associated with SDAI disease activity during MTX identified another parameter such as folic acid supplementation (Fig) [24]. Those taking 1 mg/d, but not 10 mg/wk, were found to have higher disease activity during 24 weeks of MTX monotherapy, favoring folic acid supplementation 10 mg weekly over 1 mg daily. Meanwhile, MTX-PG and glucocorticoid use were found to be associated with lower SDAI (Fig), consistent with the results proposed by the population PK/PD modeling [9]. There are potential issues when we consider applying the measurements of erythrocyte MTX-PGs in the management of RA in clinical practice (Table 2).

**Figure:**
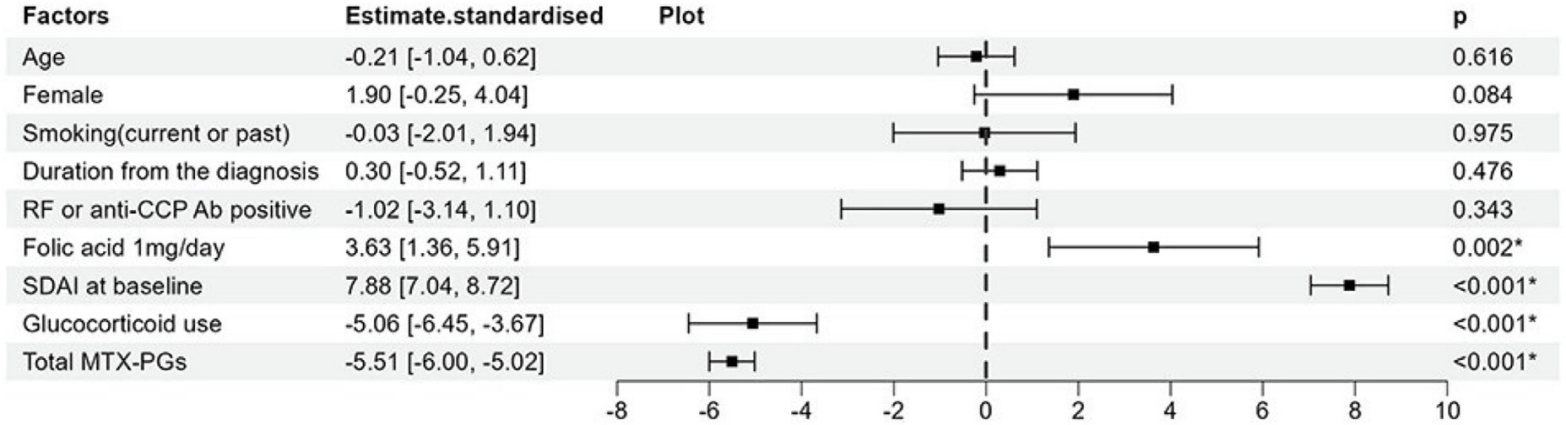
Mixed-effects models for repeated measure analysis for SDAI during 24-week MTX monotherapy.

**Table 2:** Monitoring of MTX-PG concentrations in RBCs

• Cost may be high. • Technique is sophisticated (LC-MS/MS with expensive internal controls). • Report is taking several days to weeks. • Cutoff for efficacy and safety needs further studies to make a consensus. • It provides a great insight into our understanding and theoretical explanation of the results of clinical trials/studies.

MTX-PG, methotrexate-polyglutamate; RBC, red blood cell.

## bDMARD DRUG LEVELS

Recent randomized clinical trials comparing TDM for infliximab versus standard therapy without TDM have clearly demonstrated that monitoring infliximab drug levels is not useful for induction therapy [25], but it may be beneficial in the chronic maintenance phase [26]. Nevertheless, the cost of TDM may be a concern in clinical practice. On the contrary, measuring drug concentrations may be helpful not only in early clinical development but also in late phase 2/phase 3 clinical trials, in which the number of enrolled patients is limited and the backgrounds of the patients such as the previous exposure to bDMARDs are so diverse, making the subgroup analysis difficult. In these situations, determining the appropriate dosage for efficacy and safety is much more difficult. Although measuring drug concentrations of bDMARDs does not completely resolve this issue, it may facilitate our understanding of how much of the drug exists in the circulating blood. For example, the kinetics of ozoralizumab drug concentrations in patients with MTX-IR RA showed a rapid increase and sustained concentrations of ozoralizumab [12]. The minimal efficacious dose could be 1 µg/L, and the 30-mg fixed dose of ozoralizumab theoretically exceeds this level as a group average [12], supporting the results of the clinical study [27]. Modeling the PK/PD of the molecule illustrated that factors such as body weight may influence the PK-PD of ozoralizumab [28]. These results with a rapid onset but longer sustainability may provide useful information for clinical practice [29]. There are several issues for the timing of measurements, quality management of measures, the appropriate cutoffs, costs, and so on. Although TDM of the bDMARDs may not be useful for determining the intervals and dosing in clinical practice in RA, they can generate hypotheses for further investigation.

## Conclusion

The role of TDM in clinical practice should be carefully evaluated, keeping in mind whether it is useful or futile in the area and scope of application to rheumatology [3,30]. On the contrary, the measuring drug levels as a biomarker may provide additional proof and supplement the interpretation of the results obtained from clinical trials and studies.

## Funding

This research did not receive any specific grant from funding agencies in the public, commercial, or not-for-profit sectors.

## CRediT authorship contribution statement

**Tsutomu Takeuchi:** Writing – review & editing, Writing – original draft, Visualization, Validation, Supervision, Software, Resources, Project administration, Methodology, Investigation, Conceptualization. **Hiroya Tamai:** Writing – review & editing, Resources, Methodology, Investigation. **Yuko Kaneko:** Writing – review & editing, Software, Resources, Methodology, Investigation.

## Competing interests

TT received consulting fees from AbbVie, EliLilly, Gilead Sciences, Mitsubishi-Tanabe, and Taisho; honoraria from AbbVie, Astellas, AstraZeneca, Chugai, Eisai, Eli Lilly, Gilead Sciences, Janssen, Mitsubishi-Tanabe, Pfizer, and Taisho. HT received honoraria from AbbVie and Eisai and support for attending meetings from PhRMA. YK received grants from AbbVie, Asahi Kasei, Ayumi, Boehringer Ingelheim, Chugai, Eisai, Gilead Sciences, Mitsubishi-Tanabe, Taisho, and UCB; consulting fees from AbbVie, Asahi Kasei, Bristol-Myers Squibb, Eli Lilly, Gilead Sciences, Pfizer, Taisho, and UCB; honoraria from AbbVie, Asahi Kasei, Astellas, Astra Zeneca, Ayumi, Bristol-Myers Squibb, Chugai, Daiichi-Sankyo, Eisai, Eli Lilly, Gilead, GlaxoSmithKline, Janssen, Mitsubishi-Tanabe, Novartis, Pfizer, Sanofi, Taisho, and UCB.
